# miRNAs regulate SIRT1 expression during mouse embryonic stem cell differentiation and in adult mouse tissues

**DOI:** 10.18632/aging.100176

**Published:** 2010-07-17

**Authors:** Laura R. Saunders, Amar Deep Sharma, Jaime Tawney, Masato Nakagawa, Keisuke Okita, Shinya Yamanaka, Holger Willenbring, Eric Verdin

**Affiliations:** ^1^ Gladstone Institute of Virology & Immunology, University of California, San Francisco, CA 94158, USA; ^2^ Department of Medicine, University of California, San Francisco, CA 94158, USA; ^3^ Institute for Regeneration Medicine, Department of Surgery, Division of Transplantation, University of California, San Francisco, CA 94158, USA; ^4^ Center for iPS Cell Research and Application, Kyoto University , Kyoto, Japan; ^5^ Gladstone Institute of Cardiovascular Disease, University of California, San Francisco, CA 94158, USA

**Keywords:** SIRT1; mouse embryonic stem cells; miRNAs; differentiation; post-transcriptional regulation; reprogramming

## Abstract

SIRT1 is increasingly recognized as a critical regulator of stress responses, replicative senescence, inflammation, metabolism, and aging. SIRT1 expression is regulated transcriptionally and post-transcriptionally, and its enzymatic activity is controlled by NAD^+^ levels and interacting proteins. We found that SIRT1 protein levels were much higher in mouse embryonic stem cells (mESCs) than in differentiated tissues. miRNAs post-transcriptionally downregulated SIRT1 during mESC differentiation and maintained low levels of SIRT1 expression in differentiated tissues. Specifically, miR-181a and b, miR-9, miR-204, miR-199b, and miR-135a suppressed SIRT1 protein expression. Inhibition of mir-9, the SIRT1-targeting miRNA induced earliest during mESC differentiation, prevented SIRT1 downregulation. Conversely, SIRT1 protein levels were upregulated post-transcriptionally during the reprogramming of mouse embryonic fibroblasts (MEFs) into induced pluripotent stem (iPS) cells. The regulation of SIRT1 protein levels by miRNAs might provide new opportunities for therapeutic tissue-specific modulation of SIRT1 expression and for reprogramming of somatic cells into iPS cells.

## Introduction

As multicellular organisms age, somatic tissues show evidence of genomic instability and an increased error rate in protein synthesis. In contrast, the germ line is protected from genomic instability to ensure the ultimate survival of its genome. As the disposable soma theory of aging suggests, maintaining a low error rate is energy intensive, so somatic cells may trade off a high level of accuracy to save energy, leading to instability and eventually error catastrophe in aging somatic cells [1]. As embryonic stem cells (ESCs) can differentiate into all cell types, including the germ line, they must expend energy to maintain the genome and repair damage. Multiple stress defense mechanisms, such as telomere maintenance, antioxidant function, and DNA repair, are highly active in ESCs and downregulated during differentiation [2].

SIRT1 protects against age-related diseases by deacetylating targets (e.g., p53, FOXO, NFκB, and PGC-1α) that regulate diverse cellular processes, including stress response, replicative senescence, inflammation, and metabolism [3, 4]. SIRT1 protein levels are high in mouse embryonic stem cells [5, 6] and participates in the defense against oxidative stress in these cells [7]. Several *Caenorhabditis elegans* genes that ensure the genomic integrity of the germ line are also involved in regulating lifespan although it is not known if this protection is conserved in higher organisms [8]. As Sir2, the *C. elegans* homolog of SIRT1, regulates lifespan [9], SIRT1 may be a gene whose high-level expression in the germ line and ESCs maintains genomic integrity and plays a key role in regulating lifespan.

SIRT1 is critical for development: loss of both SIRT1 alleles in mice leads to postnatal lethality. Mice lacking SIRT1 survive when outbred but yield smaller, sterile mice with developmental defects [10, 11]. In addition, SIRT1 expression is induced during calorie restriction (CR), a 20-40% lowering of caloric intake that extends lifespan [12]. Transgenic mice that overexpress SIRT1 partially phenocopy CR [13], and are protected from age-related diseases such as diabetes, osteoporosis, and cancer [14]. SIRT1^-/-^ mice do not have a longer lifespan on a CR diet [15]. Resveratrol, a polyphenol from grapes, works via the SIRT1 pathway to extend the lifespan of older mice fed a high-fat diet [16]. Similar to resveratrol, small-molecule activators of SIRT1 mimic the beneficial effects of CR and protect mice against age-related diseases [17, 18].

These observations highlight the importance of tightly regulating SIRT1 and the benefits of increasing SIRT1 expression and activity to promote longevity and suppress age-related diseases. Tight regulation of SIRT1 expression and activity is achieved through regulation of transcription by p53, FOXO3a, and E2F1 [19, 20]. SIRT1 expression is also regulated by controlling mRNA stability by HuR [21] and its enzymatic activity is sensitive to cellular NAD^+^ levels [22, 23] SIRT1-interacting proteins such as DBC1 and AROS also regulate its activity [24, 22].

Here we report that SIRT1 is highly expressed in mESCs compared to differentiated tissues and identify several miRNAs that regulate its expression at a post-transcriptional level during differentiation.

## Results

### SIRT1 protein is expressed at high levels in mESCs and post-transcriptionally downregulated during differentiation

**Figure 1.**
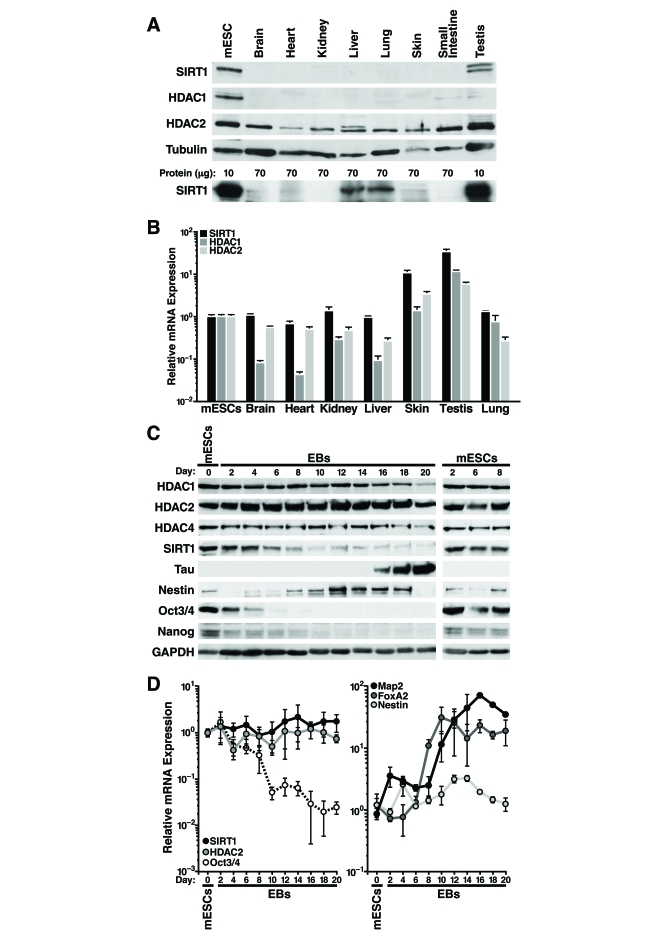
SIRT1 expression is regulated post-transcriptionally in adult mouse tissues and during mESC differentiation. (**A-B**) Protein and RNA were extracted from mESC and tissues from ~6-week-old mice. (**A**) Western blot analysis with antibodies against SIRT1 (Frye antiserum top blot; Upstate antiserum lower blot), HDAC1, HDAC2, and tubulin. (**B**) qRT-PCR analysis of SIRT1, HDAC1, and HDAC2 normalized to GAPDH levels. Data are mean ± s.d. for four samples. (**C-D**) Protein and RNA were isolated from mESCs differentiated *in vitro* for up to 20 days (EBs d2-20). (**C**) Western blots analysis of expression of SIRT1, various HDACs, markers of pluripotent embryonic stem cells, and markers of differentiation. Data are representative of four experiments. (**D**) qRT-PCR analysis of SIRT1, HDAC2, markers of pluripotent embryonic stem cells, and markers of differentiation. Data were normalized to GAPDH and plotted as expression relative to the mean of four mESC samples. Data are mean ± s.d. for four samples.

We observed that SIRT1 protein levels are higher in mESCs than differentiated mouse tissues (Figure 1A). Overloading of lysate from differentiated tissues and a different SIRT1 antibody confirmed ubiquitous expression of SIRT1 in differentiated tissues, however expression was significantly lower levels than in mESCs (Figure 1A, lower panel). HDAC1 protein levels were also higher in mESCs, whereas HDAC2 protein expression was similar in mESCs and differentiated tissues (Figure 1A). Strikingly, measurement of SIRT1 mRNA levels by quantitative reverse transcription-PCR (qRT-PCR) showed relatively similar levels in mESCs and differentiated mouse tissues, except for skin and testis where mRNA levels were significantly higher (Figure 1B). In contrast, HDAC1 and HDAC2 mRNA correlated more closely with protein expression: HDAC1 mRNA levels were much lower (5-15 fold) in most differentiated tissues than in mESCs, whereas HDAC2 mRNA levels were similar in mESCs and differentiated tissues (Figure 1B). These findings of discordant mRNA and protein levels of SIRT1 suggested that SIRT1 is regulated post-transcriptionally in most adult mouse tissues.

To determine if SIRT1 is also regulated post-transcriptionally during *in vitro *differentiation of mESCs, we removed leukemia inhibitory factor (LIF) from the culture medium to allow the cells to differentiate into embryoid bodies. Protein and RNA were isolated from the mESCs and embryoid bodies every two days during *in vitro* differentiation. At d6 of the differentiation process, the high SIRT1 protein levels found in undifferentiated mESCs began to decrease (Figure 1C). Control mESCs cultured under non-differentiating conditions showed no change in SIRT1 expression (Figure 1C, right panel). In addition, SIRT1 protein expression levels decreased during directed differentiation of mESCs into neurons (Supplementary Figure 1). HDAC1 and HDAC4 expression were high in mESCs and decreased late during differentiation with kinetics distinct from that of SIRT1 (Figure 1C). In contrast, HDAC2 protein levels remained constant during *in vitro* differentiation. As expected, markers of pluripotency, including Nanog, Sox2, and Oct-3/4, were expressed in mESCs and decreased early during differentiation (Figure 1C and data not shown). In embryoid bodies, which exhibit spontaneous neural differentiation, the neuronal precursor marker Nestin was transiently induced, whereas Tau, a marker of mature neurons, was induced at late differentiation stages (Figure 1C).

In contrast to the decrease in SIRT1 protein levels observed during *in vitro* differentiation of mESCs, SIRT1 mRNA levels showed no change (Figure 1D, left panel). HDAC2 mRNA levels mirrored protein levels and were unchanged during differentiation. mRNAs levels of pluripotent stem cell markers, including Oct-3/4 (Figure 1D, left panel), Nanog, and Sox2 (data not shown) decreased during differentiation. mRNA expression of the ectoderm marker Map2 and the endoderm marker FoxA2 increased during differentiation, and Nestin mRNA expression transiently increased (Figure 1D, right panel).

**Figure 2.**
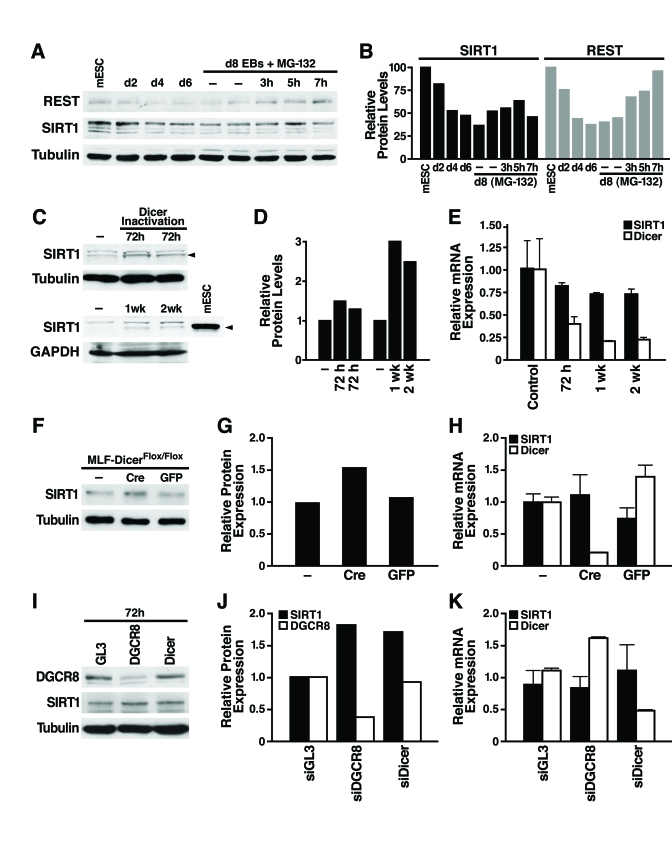
miRNAs post-transcriptionally regulate SIRT1. (**A**) mESCs were differentiated and treated on d8 with the proteasome inhibitor MG-132 (10 μM, 3-7 h), and protein lysates were analyzed on western blots. Data are representative of four experiments. (**B**) Protein levels of SIRT1 and REST relative to tubulin levels were quantified by densitometry with NIH Image. (**C-E**) The consequences of Dicer inactivation and loss of small RNAs were assessed in protein lysates and RNA from livers of control and Dicer^flox/flox^ mice injected with the AAV8 vector expressing cre at the indicated times. (**C**) Western blotting was used to analyze 70 μg of liver lysate and 10 μg of mESC lysate. (**D**) SIRT1 protein levels relative to tubulin or GAPDH were quantified by densitometry. (**E**) SIRT1 and Dicer mRNA levels were measured by qRT-PCR. Data are mean ± s.d. for four samples. (**F-H**) Lung fibroblasts were cultured from Dicer^Flox/Flox^ mice and infected with adenoviral Cre or GFP. (**F**) SIRT1 protein levels were measured by western blotting 72 h after Cre inactivation of Dicer. (**G**) SIRT1 protein levels relative to tubulin were quantified by densitometry. *(***H***)* mRNA levels of SIRT1 and Dicer were measured by qRT-PCR. Data are mean ± s.d. for three samples. (**I-K**) siRNAs were transfected into NIH3T3 cells to knockdown DGCR8, Dicer, or GL3 luciferase as a control. (**I**) DGCR8 knockdown and increased SIRT1 protein levels were analyzed by western blotting 72 h after siRNA transfection. Data are representative of three experiments. (**G**) qRT-PCR analysis confirmed Dicer knockdown and no significant change in SIRT1 mRNA levels. Data are mean ± s.d. for three samples.

### A Dicer-dependent pathway post-transcriptionally regulates SIRT1 expression

To examine the mechanism of SIRT1 post-transcriptional regulation, we first tested whether SIRT1 protein stability is controlled by the proteasome. As a positive control, we confirmed that REST, an essential protein in undifferentiated mESCs that represses neuronal genes in differentiated non-neuronal tissues, was downregulated by the proteasome during differentiation as previously reported [26]. Treatment of d8 embryoid bodies with the proteasome inhibitor MG-132 increased REST protein expression; however, in the same cell culture population, proteasome inhibition did not increase SIRT1 protein expression (Figure 2A and B; Supplementary Figure 2). Thus, proteasome-mediated degradation of SIRT1 is not responsible for its post-transcriptional downregulation during differentia-tion.

We next determined if SIRT1 is subject to post-transcriptional regulation by miRNAs [27] For this purpose, we inactivated Dicer, an enzyme required for processing of small RNAs, including miRNAs, into their mature functional form [28] We injected Dicer^flox/flox^ mice [29] with an adeno-associated viral (AAV) vector expressing Cre from the hepatocyte-specific transthyretin promoter. Liver-specific inactivation of Dicer increased SIRT1 protein levels (Figure 2C, D) while SIRT1 mRNA levels slightly decreased (Figure 2E). Additionally, we isolated lung fibroblasts from the Dicer^flox/flox^ mice and infected them with an adenovirus expressing Cre or GFP. Cre-mediated inactivation of Dicer increased SIRT1 protein levels (Figure2F, G), without changing SIRT1 mRNA levels (Figure 2H). To determine whether miRNAs or other small RNAs regulate SIRT1 in differentiated tissues, we knocked down the expression of DGCR8, which is specifically required for processing of miRNAs, and Dicer in mouse NIH3T3 cells. Knockdown of either DGCR8 or Dicer increased SIRT1 protein expression (Figure 2I, J) without changing SIRT1 mRNA levels (Figure 2K). Knockdown of Dicer was verified by qRT-PCR mRNA measurement and knockdown of DGCR8 was verified by western blot (Figure 2 I-K). Thus, miRNAs post-transcriptionally regulate SIRT1 in differentiated tissues and cell lines, and may account for the downregulation of SIRT1 during *in vitro* mESC differentiation.

### The SIRT1 mRNA 3'-UTR is targeted by multiple miRNAs

**Figure 3.**
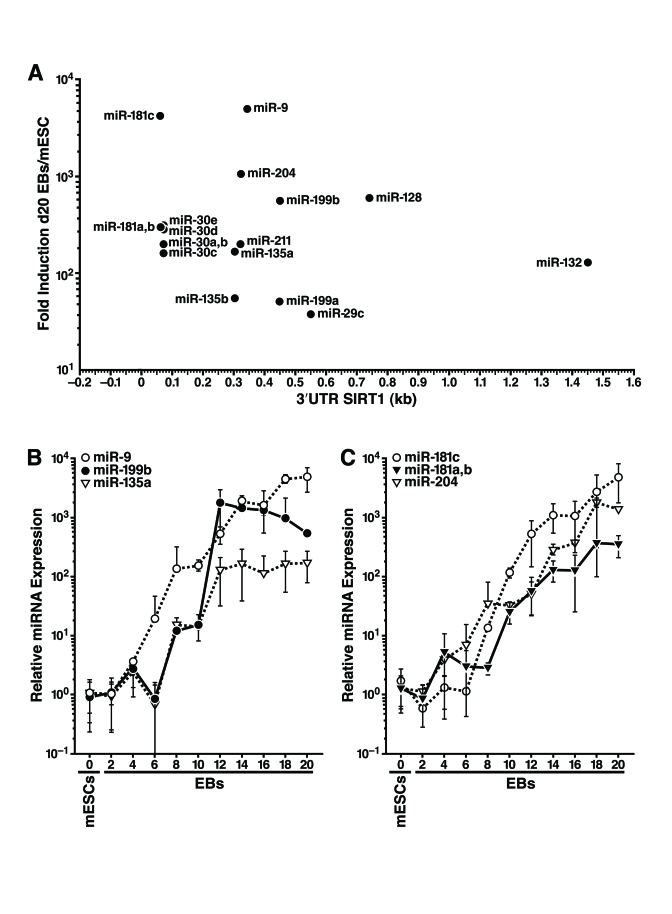
Expression profiling of miRNAs that potentially target the SIRT1 3'-UTR during mESC differentiation. (**A**) 18 miRNAs from nine miRNA families that potentially target the 3'-UTR of SIRT1 were induced during mESC differentiation at the time SIRT1 protein was downregulated. Their fold induction in d20 embryoid bodies above their expression in undifferentiated mESCs was plotted on the y-axis, and the location of their seed binding site in the 3'-UTR of mSIRT1 was plotted on the x-axis. (**B-C**), qRT-PCR of miRNA expression relative to miR-16 from undifferentiated mESCs and differentiating embryoid bodies of specific miRNAs that potentially target SIRT1. Data are mean ± s.d. for four samples.

To identify miRNAs that target SIRT1, we examined the 1.6-kb mSIRT1 3'-UTR with algorithms that predict miRNA target sites [30, 31]. Target Scan 5.1 revealed 22 miRNAs targeting 12 broadly conserved seed sites in the 3'-UTR of mSIRT1. This analysis also revealed two miRNAs targeting three seed sites conserved only in mammals, and 66 seed sites for poorly conserved miRNA families. In contrast, HDAC1, which has a shorter 3'-UTR (0.5 kb), had no broadly conserved miRNA seed sites, one seed site conserved in mammals, and 22 seed sites for poorly conserved miRNAs (data not shown). We hypothesized that if miRNAs post-transcriptionally downregulate SIRT1 during mESC differentiation, the miRNAs responsible should be induced during differentiation when SIRT1 protein levels are decreased. We used qRT-PCR to profile the expression of 39 miRNAs that potentially target SIRT1: 21 well-conserved miRNAs (representing 11 miRNA families), two miRNAs conserved only in mammals, and 16 less conserved miRNAs many of which had two target sites in the 3'-UTR of mSIRT1 (Supplementary Table 1). We found that 18 miRNAs from nine families were upregulated 30-5000 fold during mESC differentiation (Figure 3A). The expression of six selected miRNAs during mESC differentiation is illustrated in Figure 3B,C.

### miR-181a and b, miR-9, miR-204, miR-135a, and miR-199b target endogenous SIRT1

**Figure 4.**
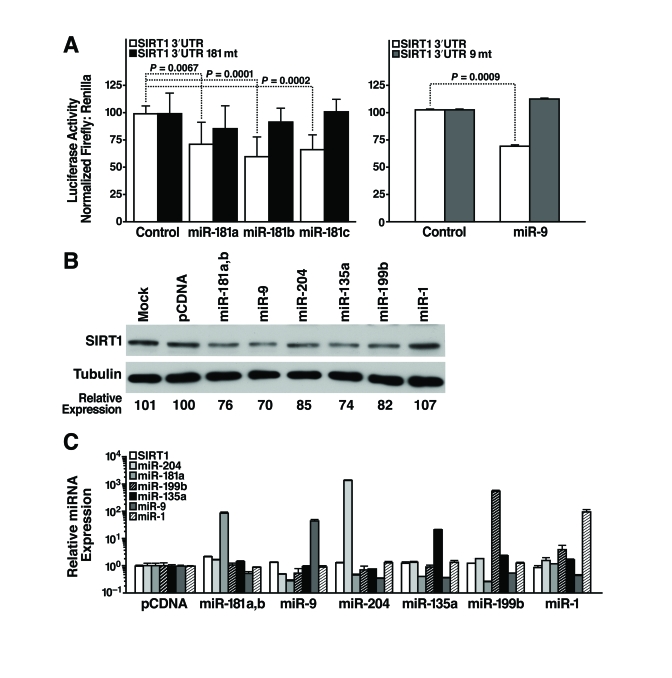
miRNAs post-transcriptionally regulate the 3'-UTR of SIRT1 mRNA. (**A**) Luciferase assays were performed 24 h after transfection of the full-length 1.6 kb SIRT1 3**'**-UTR downstream of luciferase (SIRT1 3**'**-UTR) or constructs with 4 bp in the seed-binding regions mutated (SIRT1 3**'**-UTR 181mt, left panel; SIRT1 3**'**-UTR 9mt, right panel) and control, miR-181a, b, and c miRNA mimics (left panel) or pSuper and pSuper miR-9 expression constructs (right panel). Data are mean ± s.d. for eight experiments. (**B-C**) mESCs were transfected with individual miRNA expression constructs; protein and RNA were isolated 48 h later. (**B**) Repression of SIRT1 protein was analyzed by western blotting. Data are representative of six experiments. (**C**) qRT-PCR analysis of SIRT1 mRNA levels and mature miRNA levels. Data are mean ± s.d. for four samples.

To identify miRNAs that post-transcriptionally regulate SIRT1, we cloned the 1.6-kb mSIRT1 3'-UTR downstream of luciferase, and transfected this construct (pGL3-SIRT1 3'-UTR) into mESCs along with miRNA mimics or miRNA expression constructs, and measured luciferase activity 24 h later. We found that miR-181a, b, and c repressed luciferase activity by 25-30% (Figure 4A, left panel). The specificity of this inhibition was demonstrated by testing the effect of the same miRNAs on a construct in which the miR-181 seed-binding site was mutated (pGL3-SIRT1 3'-UTR 181mt; Figure 4A, left panel). Likewise, co-transfection of a miR-9 expression vector repressed luciferase activity of pGL3-SIRT1 3'-UTR by 30% but not pGL3-SIRT1 3'-UTR 9mt, a control construct with a mutated miR-9 binding site (Figure 4A, right panel). Thus, miR-181 family members and miR-9 target the 3'-UTR of SIRT1 through the predicted seed sites.

To directly confirm the ability of select miRNAs to target the 3'-UTR of endogenous SIRT1, candidate miRNAs were introduced into mESCs and SIRT1 protein levels were assessed. Overexpression of miR-181a and b, miR-9, miR-204, miR-135a, and miR-199b decreased SIRT1 protein levels in mESCs (Figure 4B). In contrast, overexpression of miR-1, a miRNA not predicted to target the SIRT1 3'-UTR, did not decrease SIRT1 protein levels (Figure 4B). SIRT1 mRNA levels did not change upon miRNA overexpression, and the expression of individual  miRNAs  did  not  alter expression of other miRNAs (Figure 4C). These data confirm that miR-181a and b, miR-9, miR-204, miR-135a, and miR-199b target endogenous SIRT1 and downregulate its expression.

### Inhibition of miR-9 prevents the downregulation of SIRT1 protein expression during differentiation

**Figure 5.**
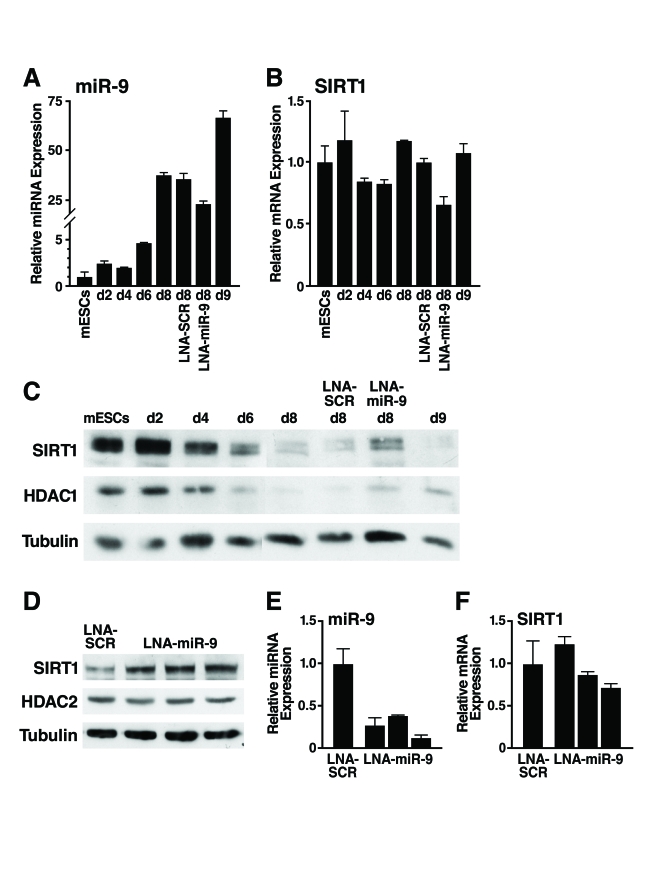
Inhibition of miR-9 prevents downregulation of SIRT1 during mESC differentiation. (**A-C**) mESCs were differentiated and transfected at d4 and d7 with LNA probes. Protein and RNA were isolated on indicated days. (**A**) qRT-PCR of miR-9 shows the expected upregulation during differentiation and 35% inhibition when embryoid bodies were transfected with LNA-miR-9 but not with LNA-SCR. (**B**) qRT-PCR show no significant change in SIRT1 mRNA levels. Data are mean ± s.d. for four samples and representative of three experiments. (**C**) Western blot analysis shows that the downregulation of SIRT1 protein during mESC differentiation was specifically inhibited in cells transfected with LNA-miR-9 but not by transfection of LNA-SCR or untransfected controls. Data are representative of four experiments. *(***D-F***)* EBs were dissociated and transfected at d6 with LNA probes. Protein and RNA were isolated on d11. (**D**) Western blot analysis shows upregulation of SIRT1 protein in EBs transfected with LNA-miR-9 but not LNA-SCR. (**E**) qRT-PCR analysis shows inhibition of miR-9 in EBs transfected with LNA-miR-9, but not with LNA-SCR, and no significant change in SIRT1 mRNA levels *(***F***).* Data are mean ± s.d. for four samples.

We consistently observed that miR-9 was the first SIRT1-targeting miRNA to be upregulated both during differentiation of mESCs into embryoid bodies (Figure 3B) and during the directed differentiation of mESCs into neurons (data not shown). miR-9 is expressed in the brain, induced during differentiation of neuronal precursors into neurons, and regulates neural lineage differentiation [32]. To confirm that miR-9 represses SIRT1 early  during mESC differentiation,  we  tested whether inhibition of miR-9 prevents the downregulation of SIRT1 protein. We used a FITC-labelled locked nucleic acid (LNA)-probe antisense to miR-9 to block miR-9 activity (LNA-miR-9). LNA-miR-9 or a scrambled control (LNA-SCR) was transfected into embryoid bodies at d4 and d7. Only cells on the outer layer of the embryoid bodies were transfected by this method, and fluorescence microscopy estimated that ~35% of cells were FITC^+^ (data not shown). As expected, miR-9 expression strongly increased during differentiation (Figure 5A). LNA-miR-9 reduced expression of miR-9 by 35% atday 8, but LNA-SCR did not. Neither inhibitor significantly altered SIRT1 mRNA expression (Figure 5B). Importantly, LNA-miR-9, but not LNA-SCR or untransfected controls, specifically prevented the differentiation-associated repression of SIRT1 protein (Figure 5C). Thus, of the 17 miRNAs upregulated during mESC differentiation that potentially target SIRT1, miR-9 acts early during differentiation to downregulate SIRT1 expression.

**Figure 6.**
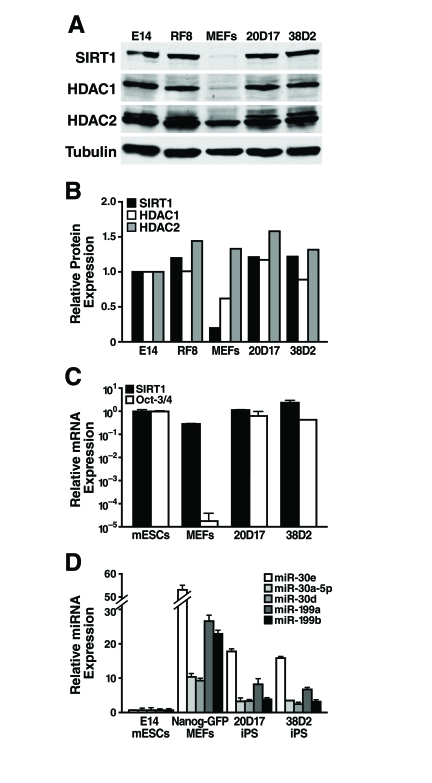
SIRT1 protein levels are upregulated during reprogramming. (**A**) mESCs, MEFs, and iPS cells were subject to western blot analysis with antibodies to the indicated proteins. *(***B***)* SIRT1, HDAC1, and HDAC2 protein levels relative to tubulin were quantified by densitometry. (**C**) qRT-PCR analysis of SIRT1 mRNA levels in mESCs, MEFs, and iPS cells were measured relative to GAPDH. (**D**) qRT-PCR analysis of miRNA expression relative to miR-16 in mESCs, MEFs, and iPS. Data are mean ± s.d. for three samples.

To enhance the fraction of cells transfected, we dissociated d6 embryoid bodies, transfected them with LNA-miR-9 or LNA-SCR, reaggregated the embryoid bodies, and assessed SIRT1 expression at d11. With this method, 70-80% of the cells in the embryoid bodies were transfected, and LNA-miR-9 specifically increased SIRT1 protein levels ~two-fold (Figure 5D) qRT-PCR analysis demonstrated a more efficient repression of miR-9 expression in the LNA-miR-9 treated cells (Figure 5E), with minimal change in SIRT1 mRNA levels (Figure 5F). These observations confirmed that miR-9 inhibition increased SIRT1 protein levels.

### SIRT1 protein levels increase during reprogramming

As SIRT1 protein levels are lower in differentiated tissues than in mESCs, we next asked if SIRT1 protein levels increase during reprogramming of mouse embryonic fibroblasts (MEFs) into induced pluripotent stem (iPS) cells. We used previously described iPS cell lines derived from retroviral mediated expression of Oct-3/4, Sox2, Klf4, and c-myc in MEFs. These iPS cell lines also express a Nanog-GFP reporter [33]. Protein levels of SIRT1 were low in the starting MEFs and were dramatically upregulated in iPS clones, to the same levels seen in two mESC lines, E14 and RF8 (Figure 6A, B). Similarly, low levels of HDAC1 protein were upregulated during reprogramming of MEFs into iPS, while HDAC2 protein levels were broadly similar in MEFs, iPS, and mESCs (Figure 6A, B). Comparison of SIRT1 mRNA levels in mESCs, MEFs, and iPS clones showed that the starting MEFs had only 30% of the SIRT1 mRNA, but this only partially explains the 6.5-fold difference in SIRT1 protein expression (Figure 6C). Thus, post-transcriptional regulation of SIRT1 contributes significantly to the upregulation of SIRT1 protein levels during reprogramming.

To identify miRNAs that may post-transcriptionally upregulate SIRT1 protein during reprogramming, expression levels of miRNAs that potentially target SIRT1 were compared in mESCs, MEFs, and iPS cells. As previously discussed, miR-199a and b were strongly upregulated during mESC differentiation (Figure 3). As predicted, reprogramming of MEFs into iPS cells was accompanied by a downregulation of miR-199a and b by 3.3-fold and 5.8-fold, respectively (Figure 6D). Additionally, all five members of the miR-30 family that potentially target SIRT1 were higher in MEFs than iPS and mESCs. Therefore, expression of select miRNAs, including the miR-199 and miR-30 families, decreases during reprogramming and may allow for the upregulation of SIRT1 protein expression.

## Discussion

Our work shows that SIRT1 is highly expressed in mESCs and that miRNAs post-transcriptionally downregulate SIRT1 protein expression during mESC differentiation and maintain low SIRT1 protein levels in differentiated adult mouse tissues. Specifically, SIRT1 expression is repressed by miR-181a and b, miR-9, miR-204, miR-135a, and miR-199b.

Repression of SIRT1 protein expression by miRNAs may play an important role in development since several miRNAs that target SIRT1 have previously been identified as regulators of specific differentiation pathways. For example, miR-9, a miRNA expressed early during mESC differentiation, participates in neuronal differentiation [32]. Since activation of SIRT1 in neuronal precursors promotes astrocyte formation over neurogenesis [34], SIRT1 might represent a critical target for miR-9. Another similar example is miR-181, which is transiently upregulated during muscle differentiation [35]. SIRT1 inhibition induces premature differentiation of C2C12 myoblasts, and SIRT1 activation inhibits muscle differentiation [36]. Thus, regulation of SIRT1 by miR-181 might contribute to the muscle differentiation program. miR-181a also regulates T-cell-receptor sensitivity and signal strength during T-cell development, in part by targeting tyrosine phosphatases [37]. Since SIRT1 inhibition induces T-cell hyperactivation [38], miR-181a may also target SIRT1 during T cell development.

Because each miRNA targets only one site in the SIRT1 3'-UTR, multiple tissue-specific miRNAs likely work together to regulate SIRT1 expression. Additionally, miRNA regulation of SIRT1 might be influenced by HuR [39], which binds the 3'-UTR and stabilizes the SIRT1 transcript [21], even though HuR binding sites do not directly overlap miRNA seed-binding sites in the SIRT1 3'-UTR. HuR, whose expression decreases during aging, is targeted by miR-519, which triggers senescence and represses tumor growth through downregulation of HuR [40, 41]. Tissue-specific therapeutic targeting of miRNAs that regulate SIRT1 might allow the selective upregulation of SIRT1 in unique tissues, whereas current small molecules that activate SIRT1 do so in a tissue non-specific manner.

We also tested whether SIRT1 protein levels might increase upon reprogramming of MEFs into iPS cells. Remarkably, we found that low SIRT1 protein levels in MEFs were upregulated during reprogramming into iPS cells to levels similar to mESCs (Figure 6A). This correlated with the downregulation of miR-199 andmiR-30 families that target SIRT1 (Figure 6C). Expression of miR-199a and b is highest in skin (Supplementary Figure 3), and limiting the expression of these specific miRNAs may be a prerequisite for reprogramming of MEFs. Reprogramming of other differentiated cell types may require downregulation of distinct tissue-specific miRNAs that regulate SIRT1 expression.

An important area for future focus will be to understand why SIRT1 protein levels are exceptionally high in mESCs. SIRT1 might be required to maintain a unique chromatin state in ESCs, or to deacetylate non-histone targets that are essential for early development. For example, SIRT1 deacetylates HSF1 to enhance its activity [42], and maternal HSF1 is required for development beyond the zygote stage [43]. Therefore, high expression of SIRT1 may work together with HSF1 during early development.

However, SIRT1 is not absolutely required during early development. Loss of SIRT1 on an outbred genetic background allows for 50% of SIRT1^-/-^ mice to develop relatively normally [11]. Importantly, other SIRT1 null mouse models show that SIRT1^-/- ^mice are not obtained at expected ratios with the majority of SIRT1^-/-^ mice dieing right after birth [10] or between E9.5 and E14.5 [44]. It is possible that another deacetylase, namely HDAC1, which is also both highly expressed in mESCs (Figure 1A) and upregulated during reprogramming (Figure 6A), partially compensates for SIRT1. In support of this idea, many non-histone targets are deacetylated by both SIRT1 and HDAC1 including p53 and NF-κB [4].

At least in lower organisms, SIRT1 regulates lifespan, and several genes that regulate lifespan also maintain genomic integrity in germ cells and stem cells [8]. A possible role of SIRT1 in mESCs and during early development could be to monitor quality control of developing embryos. SIRT1 may respond to oxidative stress, genotoxic damage, metabolic defects, and epigenetic reprogramming errors, possibly through the deacetylation of p53 and other targets, to regulate survival of developing embryos. Indeed, expression level and activity of p53 in early pre-implantation embryos regulates their viability [45].

Another intriguing question is whether downregulation of SIRT1 is necessary during differentiation and development. SIRT1 may be downregulated during differentiation in a manner similar to other stress defense mechanisms that are highly active in ESCs [2]. The downregulation of SIRT1 via a post-transcriptional mechanism allows its mRNA to persist and might allow SIRT1 expression to be rapidly induced during stress when energy intensive cell repair and survival mechanisms are required. The decrease of SIRT1 protein levels observed during aging may conserve energy but may also contribute to increased genomic instability [46].

Loss of miRNAs might contribute to the overexpression of SIRT1 in cancer. For example, loss of miR-34a leads to SIRT1 overexpression in cancer [47, 48]. Some results point to direct binding of miR-34a to the SIRT1 3'-UTR whereas others have suggested indirect regulation of SIRT1 by miR-34a [47, 48]. Several other miRNAs that target SIRT1 are lost in cancers. For example, miR-181a and b function as tumor suppressors in the brain, but their loss negatively correlates with glioma grade, and restoration of their expression induces apoptosis of glioma cells [49]. Furthermore, miR-181 and miR-29 family members are downregulated in chronic lymphocytic leukemia, and miR-29 is lost in colon, breast, and lung cancer [50, 53]. While SIRT1 may function as a tumor suppressor by limiting replicative senescence in primary cells, SIRT1 overexpression is seen in many cancers where it may promote cell survival [4]. Reintroduction of miRNAs lost in cancers that overexpress SIRT1 may be of therapeutic value against cancers dependent on the overexpression of SIRT1.

Our findings that miRNAs regulate SIRT1 expression suggest that inhibiting specific miRNAs may be of therapeutic value in disease conditions where SIRT1 activity has been shown to be beneficial such as diabetes, neurodegeneration, and cancer [54]. Currently available small molecule SIRT1 activators and inhibitors globally increase or inhibit SIRT1 activity. In contrast, the use of tissue-specific miRNA mimics or inhibitors may allow for the tissue-specific regulation of SIRT1 to prevent and treat age-related diseases without globally altering SIRT1 activity.

## Methods

Culturing and differentiation of mESCs. E14 mESCs [55] were cultured feeder-free in Glasgow MEM/BHK12 (GMEM; Sigma-Aldrich; St. Louis, MO) supplemented with 10% characterized fetal bovine serum (FBS; Hyclone; Logan, UT), 2 mM L-glutamine (GIBCO Invitrogen Corporation; Carlsbad, CA), 1 mM sodium pyruvate (GIBCO Invitrogen Coroporation), 0.5 mM β-mercaptoethanol (Sigma), and leukaemia inhibitory factor (LIF) conditioned medium on plates coated with 0.1% bovine gelatin (Sigma) in PBS. Undifferentiated ESCs were passaged every 2 days, and medium was changed on alternate days. Differentiation was induced by plating 3x10^6^ cells in 10-cm, ultra-low attachment dishes (Corning; Lowell, MA) in 10 ml of differentiation medium (GMEM supplemented with 15% FBS, 2 mM L-glutamine, 1 mM sodium pyruvate, and 0.5 mM β-mercaptoethanol). Medium on the embryoid bodies was changed every 2 days. The proteasome inhibitor MG-132 (10 μM; Calbiochem; Darmstadt, Germany) was added to the media of d8 embryoid bodies for the indicated times.

For neuronal differentiation, 5 μM retinoic acid (Sigma R-2625) was added to d4 embryoid bodies [56]; then d8 embryoid bodies were trypsinized to form a single-cell suspension. Cells were strained through a 40-μM nylon mesh (BD Biosciences; San Jose, CA), and 8x10^5^ cells in 1 ml of neurobasal A (NBA) medium (Invitrogen) supplemented with 2% B27 supplement (Invitrogen) and 500 μM glutamine were plated onto poly-D-lysine/mouse laminin 12-mm coverslips (BD Biosciences) in 24-well plates. Medium was changed 2 and 24 h after plating. After 2 days, the medium was changed to NBA supplemented with 1% N2 (Invitrogen) and 500 μM glutamine.

Expression constructs. The full-length 1.6 kb mSIRT1 3'-UTR was PCRed from IMAGE clone 3587177 (Open Biosystems; Huntsville, AB) with primers that add NheI sites (underlined): forward, 5'-TCATAACGCTAGC GA AGCTGTCCG-3'; reverse, 5'-TCCAGTCATTAAACG GGCTAGC AAAC-3'. This SIRT1 3'-UTR was cloned behind luciferase in the pGL3-promoter vector (Promega; Madison, WI) digested with XbaI. Site-directed mutagenesis was performed using a QuikChange II Site-Directed Mutagenesis kit (Stratagene; La Jolla, CA) to mutate base pairs 3-6 in the predicted seed region targeted by miR-181 and miR-9 in the SIRT1 3'-UTR.

Genomic DNA 250-350 bp on either side of the genomic locus for miR-181a and b, miR-9, miR-204, miR-135a, and miR-199b was amplified and cloned into pCDNA/V5-DEST (Invitrogen) with the following primers: mmu-miR-181a and mmu-miR-181b amplified from chromosome 1 (5'-CACCAACAGCCTGTAACT AAGCTCC-3' and 5'-TGATTCTGGGCATCCAACAC -3'), mmu-miR-9-2 amplified from chromosome 13 (5'-CTAGCCGCACACACTAAG-3' and 5'-TGCATCCCA CTTTCAATCATA-3'), mmu-miR-204 amplified from chromosome 19 (5'-CACCTTCATTCAGCACCTAGT TGAG-3' and 5'-ATACATTACAACCTGTTCAGAGG -3'), mmu-miR-199b amplified from chromosome 2 (5'-CCACAGGAGGCAGAAGGGGAGTCG-3' and 5'-CCCATCAGCCCAGCCATTTGC-3'), and mmu-miR-135a amplified from chromosome 9 (5'-CACCTCAG TGTCCAATGGGAATAC-3' and 5'-GGCTATCAAGG GGTTTCTTCAGG-3'). miR-1 was cloned as described [57].

Western blot analysis .  mESCs, Embryoid bodies, and neurons were lysed in 50 mM Tris-HCl (pH 7.5), 0.5 mM EDTA, 150 mM NaCl, 0.5% NP-40, and 1x complete protease inhibitors (Roche; Penzberg, Germany), and protein concentrations were determined with the D_C_ Protein Assay (Bio-Rad). Organs harvested from ~6-week-old mice were lysed (0.1 g/ml) in 50 mM Tris-HCl (pH 7.5), 0.5 mM EDTA, 500 mM NaCl, 0.5% NP-40, and 1x complete protease inhibitors (Roche) with a Dounce homogenizer. Protein samples were separated by electrophoresis on 7.5% or 10% SDS-polyacrylamide gels and transferred to nitrocellulose membranes (Bio-Rad). Membranes were blocked with 5% nonfat dry milk in TBS-Tween [10 mM Tris-HCl (pH 7.5), 150 mM NaCl, and 0.1% Tween-20] and probed with antiserum against HDAC1 [58], HDAC2 (Santa Cruz #7899), SIRT1 (polyclonal antiserum to amino acids 506-747 of hSIRT1 or Millipore #07-131), GAPDH (Novus Biologicals; Littleton, CO), Actin (Sigma), HDAC4 [59], Tau (EMD Biosciences; Germany), Nestin (Millipore; Billerica, MA), Oct-3/4 (R&D Systems), Nanog (Cosmo Bio; Tokyo, Japan), REST (Millipore), DGCR8 (Proteintech; Chicago, IL) and α-tubulin (Sigma).

Quantitative RT-PCR. Total RNA was isolated using TRIzol (Invitrogen). With Superscript II or III (Invitrogen) and oligo dT, 1 μg of RNA was reverse transcribed into cDNA. with Superscript II or III (Invitrogen) and oligo dT. Relative expression levels were determined by real-time quantitative PCR in an ABI 7700 or 7900 and normalized to GAPDH. 2X HotSybr Real-time PCR mix (McLab; South San Francisco, CA) was used with validated primers for HDAC1 (PPM04372A), HDAC2 (PPM04361A), and SIRT1 (PPM05054A; SuperArray Bioscience; Frederick, MD). GAPDH was amplified using (forward: 5'-ACTCCACT CACGGCAAATTCA, reverse: 5'-GCCTCACCCCATT TGATGTT), Oct-3/4 was amplified using (forward: 5'- TCAGCCTTAAGAACATGTGTAAGC, reverse: 5'- GTCTCCGATTTGCATATT CTCC), and Dicer was amplified using (forward 5'- TGGGAGATGCGATTT TGGA, reverse: 5'- GCTGCCC GTGGGTCTTCATAA). 2X HoTaq Real-time PCR mix (McLab) was used with validated primers from Applied Biosystems for Nestin (Mm00450205_m1), SIRT1 (Mm_00490758_m1), FoxA2 (Mm01976556_ s1), and Map2 (Mm00485230_m1).

Relative miRNA expression levels were quantified using the NCode miRNA first-strand cDNA synthesis kit (Invitrogen) to add a polyA tail onto the miRNAs. qPCR was performed using a forward primer to the exact sequence of the target miRNA and a reverse primer provided in the NCode kit. cDNA and qPCR reactions were generated using validated primers (Applied Biosystems) for hsa-miR-16 (4373121), has-miR-181a (4373117), hsa-miR-9 (4373285), has-miR-204 (4373313), has-miR-199b (4373309), has-miR-135a (4373140), and hsa-miR-1 (4395333).

AAV8 vector preparation and Adenovirus adenovirus infection. The double-stranded AAV8 vector for the expression of *Cre* from the transthyretin promoter was described (Amar Deep Sharma et al., manuscript submitted). Briefly, A293 cells were transfected with the AAV vector plasmid, the adenoviral helper plasmid pAd5, and the AAV8 capsid expression plasmid p5E18-VD2/8 [60] by the calcium phosphate method. Virus was collected 72 h after transfection and concentrated by centrifugation on cesium chloride density gradients. Viral titer was determined by dot blot analysis. Viral particles (2 x 10^11^ in 100 μl) were injected into the tail vein of Dicer^flox/flox^ mice [11]. Livers were harvested 72 h, 1 wk, and 2 wk after virus injection.

Lungs from Dicer^flox/flox^ mice were cut into small pieces and adhered to tissue culture plates in DMEM. Fibroblasts that grew out of the explants were collected and 80,000 lung fibroblasts were seeded in 1ml of DMEM into a 12-well plate. 24 h later, adenovirus expressing GFP or Cre was added at an MOI=100 to 500 μl of fresh DMEM in each well. Protein and RNA were isolated 72 h later.

siRNAs, miRNA mimics, and LNA probes. 20,000 NIH3T3 cells were plated per well of a 12-well plate in 1 ml of DMEM with 10% bovine calf serum without antibiotics 24 h before transfection. siGENOME SMARTpool siRNAs (10 nM) against DGCR8, Dicer, or GL3 luciferase (Thermo Scientific) were added to 100 μl of OptiMem. Lipofectamine RNAiMax (2 μl) (Invitrogen) in 98 μl of OptiMem was mixed with the siRNA for 20 min. This 200-μl solution was added along with 800 μl of fresh medium to each well. Protein and RNA were isolated at indicated time points.

miRNA mimics in the form of siRNA duplexes (Thermo Fisher Scientific; Waltham, MA) for mmu-miR-181a (C-310047-04), mmu-miR-181b (C-310182-05), mmu-miR-181c (C-310183-02), the microRNA mimic negative control (CN-001000-01), and FITC-conjugated miRCURY LNA knockdown probes (Exiqon; Woburn, MA) antisense to mmu-miR-9 (LNA-miR-9; 139459-04) or scramble control (LNA-SCR; 199002-04) were transfected into mESCs or embryoid bodies using lipofectamine 2000 (Invitrogen). Embryoid bodies were transfected by trypsininzing embryoid bodies to single-cell suspensions. 700,000 cells in 600 μl of medium were added to complexes containing 4 μl of the 25 μM LNA probe and 5 μl Lipofectamine 2000 in 300 μl of OptiMem. The cells were plated in 24-well ultra-low-attachment plates, and after 30 min, 750 μl of medium was added.

mESCs (2.5x10^5^ in 300 μl of medium) were added to complexes containing 1.6 μg of pCDNA miRNA expres-sion vectors and 3 μl of Lipofectamine 2000 (Invitrogen) in 150 μl OptiMem. The cells were plated on gelatinized 12-well plates, and 1.5 ml of medium was added after 30 min, and medium was changed the next day.

Luciferase assays. mESCs (150,000 in 1 ml of medium) were added to gelatinized 24-well plates and immediately transfected using 1 μl Lipofectamine 2000 (Invitrogen) with 20 ng Renilla luciferase as an internal control, 200 μg pGL3-SIRT1 3'-UTR or vectors with mutated seed sites, and 20 pmol (~300 ng) of the miRNA mimics or 200 ng of an miRNA expression construct. After 24 h, cells were washed in 1X PBS, lysed at room temperature for 15 min in 100 μl of 1X passive lysis buffer (Promega), and 20 μl of the lysate was used in a dual luciferase assay (Promega) in a Monolight 2010 luminometer (Analytical Luminescence Laboratory; San Diego, CA). Results were normalized to Renilla and are shown relative to samples cotransfected with a negative control miRNA or empty miRNA expression vector.

## Supplementary data

Supplementary Figure 1 SIRT1 protein is down-regulated during directed differentiation of mESCs into neurons. Western analysis of SIRT1, REST, HDAC2, Oct-3/4, and Tau during directed differentiation of mESCs into neurons by treatment with retinoic acid and plating on poly-D-lysine/laminin coated plates.

Supplementary Figure 2 SIRT1 is not post-transcriptionally regulated by the proteasome during mESC differentiation. d8 EBs were treated for the indicated times with the proteasome inhibitor MG-132 and analyzed by western blotting for expression of REST, SIRT1, and tubulin.

Supplementary Figure 3 miR-199 is highly expres-sed in mouse embryonic fibroblasts and skin. qRT-PCR analysis of miR-199a and b expression relative to miR-16 in mESCs, MEFs, and various mouse tissues. Data are mean ± s.d. for four samples.

Supplementary Table 1 miRNAs that potentially target SIRT1.
