# Supplementary figures and images for: miRNAs regulate SIRT1 expression during mouse embryonic stem cell differentiation and in adult mouse tissues
Source: Aging (Albany NY). 2010 Jul 17;2(7):415–31. doi: 10.18632/aging.100176 (PMC2933889; doi:10.18632/aging.100176)

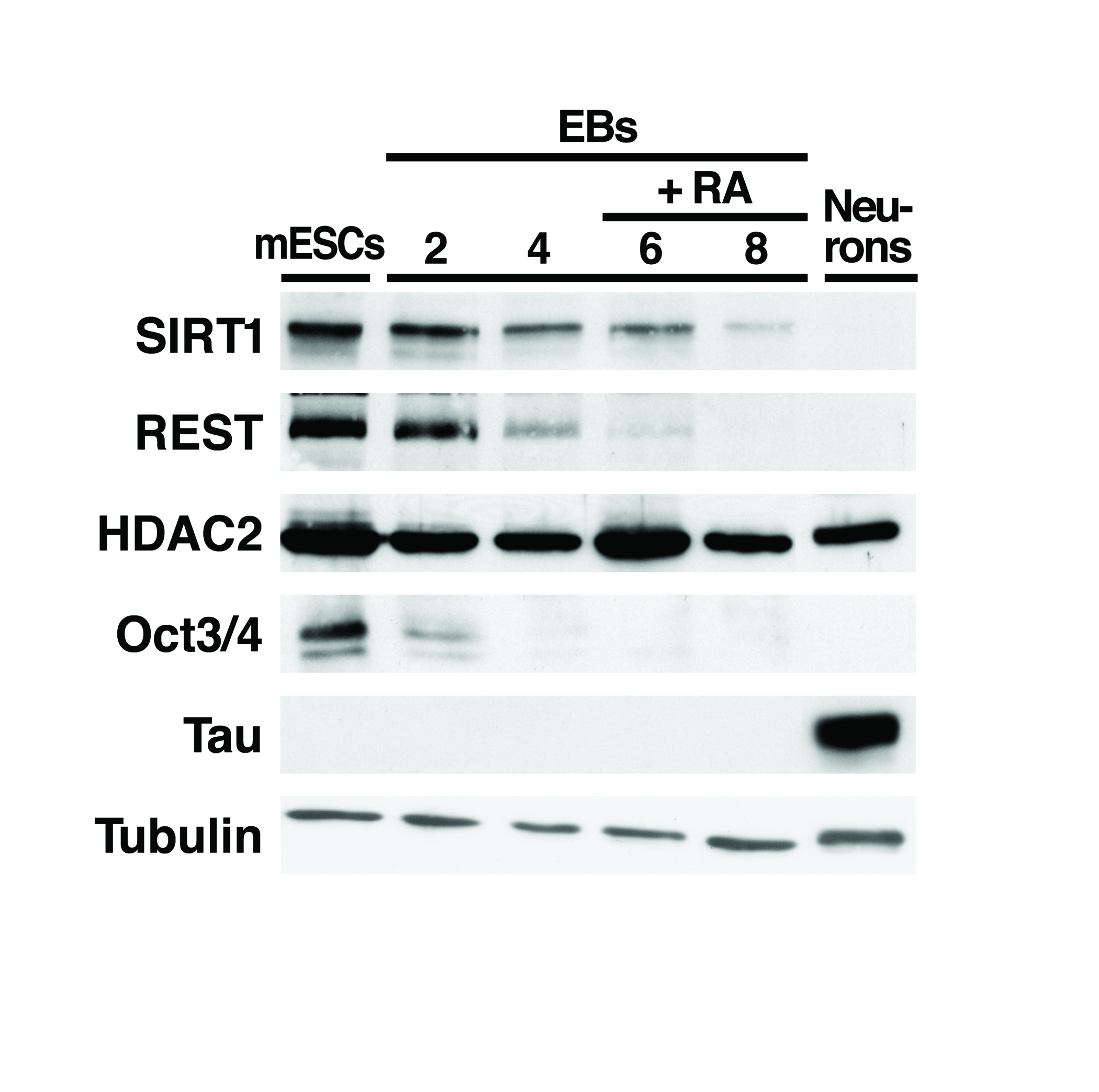

Supplement: Supplementary Figure 1 — Western analysis of SIRT1, REST, HDAC2, Oct-3/4, and Tau during directed differentiation of mESCs into neurons by treatment with retinoic acid and plating on poly-D-lysine/laminin coated plates. [file aging-02-415-s001.tif]

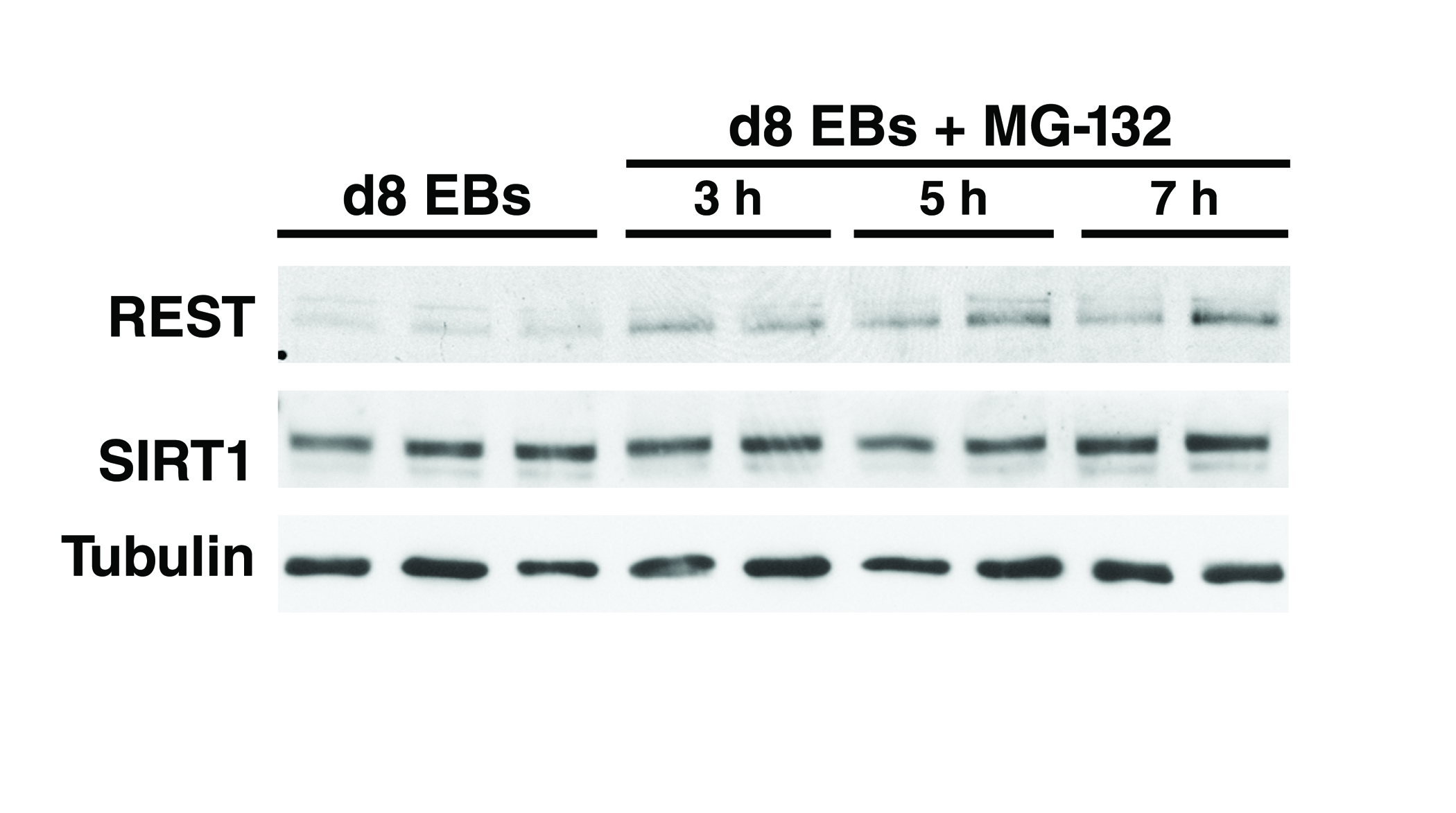

Supplement: Supplementary Figure 2 — d8 EBs were treated for the indicated times with the proteasome inhibitor MG-132 and analyzed by western blotting for expression of REST, SIRT1, and tubulin. [file aging-02-415-s002.tif]

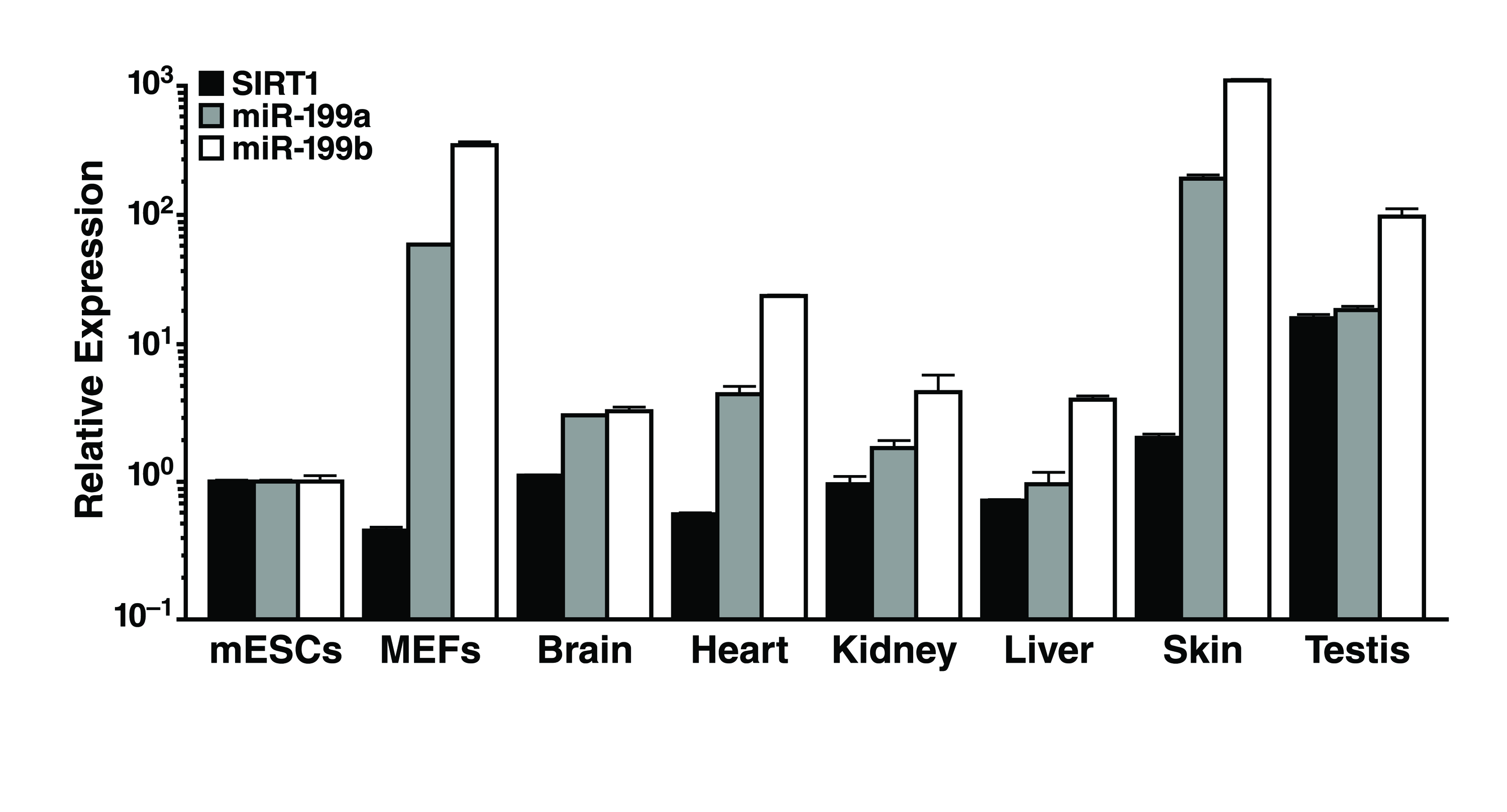

Supplement: Supplementary Figure 3 — qRT-PCR analysis of miR-199a and b expression relative to miR-16 in mESCs, MEFs, and various mouse tissues. Data are mean ± s.d. for four samples. [file aging-02-415-s003.tif]
