# Supplementary material for: miRNAs regulate SIRT1 expression during mouse embryonic stem cell differentiation and in adult mouse tissues
Source: Aging (Albany NY). 2010 Jul 17;2(7):415–31. doi: 10.18632/aging.100176 (PMC2933889; doi:10.18632/aging.100176)
Supplement: Supplementary Table 1 [file aging-02-415-s001.doc]

| **Supplementary Table 1. miRNAs that potentially target SIRT1** | | | | |
| --- | --- | --- | --- | --- |
| miRNA | Seed binding  site(s) in  3’UTR (bp) | Target Scan  Context  Score | PicTar  Probability | Fold  Upregulation  d20 EB/mESC |
| miR-9 | 345-351 | 95 | 0.97 | 5000 |
| miR-22 | 475-481 | 99 | 0.95 | <2 |
| miR-29a | 549-555 | 54 | - | 4 |
| miR-29b | 549-555 | 64 | - | <2 |
| miR-29c | 549-555 | 65 | - | 30 |
| miR-30a-5p | 72-78 | 74 | 0.67 | 180 |
| miR-30b | 72-78 | 72 | 0.67 | 180 |
| miR-30c | 72-78 | 72 | 0.67 | 160 |
| miR-30d | 72-78 | 74 | 0.67 | 280 |
| miR-30e | 72-78 | 81 | 0.84 | 300 |
| miR-34a | 781-787, 1277-1283 | 42, 38 | - | <2 |
| miR-34c | 781-787, 1277-1283 | 38, 38 | - | <2 |
| miR-124a | 1068-1074 | 36 | 0.96 | 5 |
| miR-128 | 744-750 | 84 | 0.92 | 600 |
| miR-129-5p | 61-67, 1217-1223 | 37, 64 | - | <2 |
| miR-132 | 1450-1456 | 86 | 0.74 | 130 |
